# Ultrasound Evaluation of the Deep Cerebral Venous System in Term and Preterm Neonates: Normal Features and Correlations with the Occurrence of Germinal Matrix/Intraventricular Haemorrhage

**DOI:** 10.3390/children12101347

**Published:** 2025-10-07

**Authors:** Adrian Ioan Toma, Leonard Năstase, Andreea Ioana Necula, Roxana Pavalache-Stoiciu, Miruna Harnagea, Eduard Gavrilă, Anca Roxana Bivoleanu

**Affiliations:** 1 Life Memorial Hospital, 010719 Bucharest, Romania; atoma@medlife.ro (A.I.T.); andreea.necula@s.utm.ro (A.I.N.); roxana.stoiciu@s.utm.ro (R.P.-S.); miruna.harnagea@s.utm.ro (M.H.); eduard.gavrila@s.utm.ro (E.G.); 2 Faculty of Medicine, University Titu Maiorescu, 040441 Bucharest, Romania; 3 Faculty of Medicine, University of Medicine and Pharmacy Carol Davila, 050474 Bucharest, Romania; 4 Neonatology Department, National Institute for Mother and Child Health “Alessandrescu-Rusescu”, 041249 Bucharest, Romania; 5 Regional Center of Public Health, 700465 Iasi, Romania; bivoleanua@cuzavodaiasi.ro

**Keywords:** terminal vein, internal cerebral vein, angle at confluence, venous drainage pattern, term, preterm neonates, germinal matrix/intraventricular haemorrhage

## Abstract

Background/Objectives: The variability in the structure of the deep cerebral venous system in neonates is increasingly recognised, as are the vascular structural factors involved in the development of the germinal matrix/intraventricular haemorrhage (GM/IVH) in premature infants. We aimed to characterise the ultrasound patterns of these veins in different categories of newborns and to assess if there is a correlation between certain patterns and angles and the presence of GM/IVH. Methods: One hundred neonates (68 at-term and 32 preterm) were included in this research. The pattern of venous drainage and the angle at the confluence between the terminal vein (TV) and internal cerebral vein (ICV) were identified on coronal sections through the anterior fontanel. The normal pattern was considered as that in which the confluence between the TV and the ICV could be identified, and the atypical pattern was considered the situation in which no confluence or terminal vein was identified. Results: There was no statistically significant difference regarding the normal or atypical venous patterns between the groups (*p* < 0.443), neither regarding the angles between TV and ICV between term and preterm neonates (*p* < 0.279—left; *p* < 0.718—right), and singletons and twins (*p* < 0.745 left; *p* < 0.418 right), or between the angles on the left and on the right in the whole group (*p* < 0.121 and the subgroups of term (*p* < 0.440) and preterm neonates (*p* < 0.092). The mean value of the angle at the confluence between the TV and the ICV on the left, was significantly lower in the premature infants with GM/IVH (124.90° vs. 137.02°; *p* = 0.012), being a good predictor for the occurrence of the lesion (AUC = 0.793; IC 95%: 0.580–1.006; *p* = 0.018), with a sensitivity of 79%, a specificity of 67%, and a cut-off value of 126.90°. In patients with GM/IVH, the angle was significantly lower on the side with the haemorrhage than on the side without haemorrhage (*p* < 0.043). Conclusions: There is no difference in the central venous pattern or angle at the confluence of the TV and the ICV between different categories of neonates. The angle at the confluence between the TV and ICV could identify the cases at risk for GM/IVH as well as the side of occurrence of the haemorrhage, offering the opportunity of developing personalised prevention strategies. The lack of an MRI comparator of these measurements limits the practical importance of this study.

## 1. Introduction

The in vivo identification of the structures of the cerebral deep venous system, both in adults and neonates, has been a constant topic of research. MRI and, in particular, MRI SWI venography represent the gold standard in the field [1,2]. By using this technique, an Italian group described the various types of deep venous systems in preterm and term neonates by using MR SWI venography [3]. The same group found out that the abnormal/atypical venous types were more frequent in premature neonates [3,4] and that the neonates with GM/IVH presented with a higher variability of the deep venous system and a narrower curvature of the thalamo-striate vein at the confluence with the internal cerebral vein [4]. This technique, though, has the disadvantage that special conditions are needed to perform this determination, including transport of a fragile neonate, monitoring, and skilful interpretation of the images.

Head ultrasound represents a good modality to identify the main cerebral structures in a neonate, being important especially in the case of the pathology of premature neonates [5]. Regarding the vascular anatomy of the brain, it has been known for a long time that Doppler ultrasonography (both colour and power Doppler) could identify the vast majority of the vascular structures in the neonatal brain [5,6]. The classic studies show that the internal cerebral vein and the septal vein can be identified in almost all patients [5,6], and the thalamostriate(terminal) terminal vein can be seen in many situations, and this is true also for the confluence between the terminal vein and the internal cerebral vein [6,7] situated around the foramen of Monro [6,7]. Also, the choroidal veins and the thalamocaudate veins can be identified by colour and power Doppler in certain conditions [5]. In addition, modern ultrasound techniques allow a better identification of the vascular structure, even of the small vessels with low-velocity flow [8].

The deep venous system of the brain assures the venous drainage of part of the subcortical white matter, the subependymal zone, the basal ganglia, the thalamus, and the choroid plexuses. It is traditionally classified into the medullary and the thalamostriate parts [5]. The medullary veins are fan-shaped vessels draining the blood from the periventricular white matter, which have three zones of convergence and, finally, form the subependymal veins [5]. The subependymal veins, named after the zone of the lateral ventricles they flow within, together with the anterior septal vein, finally empty into the two internal cerebral veins, which course in the lateral wall of the third ventricle and finally drain into the vein of Galen [9,10]. The deep cerebral venous system presents great variability concerning the trajectory of the subependymal veins and their confluence with the ICV. There are four described patterns in an adult [1] and six in term and preterm neonates [3]. Another important anatomic factor to be considered regarding the deep cerebral venous system is the course of the terminal vein and the angle at the confluence with the internal cerebral vein (the particular U-turn of the thalamostriate (terminal) vein at the point of confluence with the internal cerebral vein), which could determine an increased venous pressure upstream of the confluence [11,12].

Germinal matrix/intraventricular haemorrhage (GM/IVH) [11,13] is the main cerebrovascular lesion occurring in premature infants [11], remaining with a constant incidence despite the improvements in neonatal intensive care [11,14], and is also responsible for the main neurologic sequelae present in this age group [15]. Even if GM/IVH is not the only cerebrovascular disease in neonates [16,17,18], it is one of the most important, and it determines great mortality and morbidity, being an important prognostic factor [1,2]. The lesion is specific to the very premature neonates [19,20], but studies in term neonates have identified these lesions or their sequelae in 20/1000 cases [21].

The lesion occurs in a particular structure called the germinal matrix, a temporal structure situated especially in the region of the head of the caudate nucleus, ventrolateral to the anterior horns of the lateral ventricles [11], that contains mainly precursors of the neurons and glial cells that migrate to their final destination [11]. The pathogenesis of the lesion is rather complex, with multiple types of factors acting differently in different patients [11]. The classic medical textbooks identify several categories of factors: intravascular [11,22], vascular [12] and extravascular factors [11]. The vascular factors are represented by the particular structure of the vessels of the germinal matrix, which makes them prone to bleeding [17], together with the above-described anatomic particularities [11,12]. The immature vascular network represents “a persisting immature vascular rete” [12] with a particular fenestrated structure of the walls, which is in the process of involution during the period at risk [12]. An elegant anatomic–pathologic study demonstrated the venous origin of the GM/IVH [23], thus underlining the importance of the venous structures in the pathogenesis of the lesion.

The identification of a particular type of anatomic structure or angle at the level of the deep cerebral venous system in neonates who develop GM/IVH could lead to the early identification of patients at risk and early use of particular prevention strategies. Although Doppler ultrasound does not have the accuracy of MRI-based techniques in identifying the central venous structures in a neonate, the technique has the advantages of being easy to perform at the bedside of the patient and non-invasive, and it can be performed several times in less-stable patients [24,25]. Before the identification of the above-mentioned risk factors, we should first assess if the determination of these venous structures is feasible and what the venous patterns are in term and healthy preterm neonates.

Accordingly, the aims of our research were as follows:

- To identify to what extent the veins of the deep venous system of the brain can be identified by ultrasound in term and preterm neonates;
- To determine the normal values of the angles at the confluence between the terminal vein and the internal cerebral vein;
- To assess if there is a correlation between the previously mentioned angles or an abnormal venous drainage pattern observed in preterm neonates and the presence of germinal matrix haemorrhage.

## 2. Materials and Methods

This study was performed in the neonatology unit of Medlife Medical Park between June and August 2025. This study was conducted in accordance with the Declaration of Helsinki and approved by the Institutional Review Board (or Ethics Committee) of the Medlife Medical System (Sistemul Medical Medlife) (protocol code: 005; date of approval: 5 June 2025). Informed consent was obtained from the parents of the patients.

The study group consisted of 100 consecutive patients with gestational ages between 30 and 39 weeks. The inclusion criteria were delivery during the period of study, informed consent of the family present, and availability for head ultrasound between 48 and 72 h of life.

The exclusion criteria were being born out of the period of study, the presence of congenital malformations and chromosomal anomalies incompatible with life, and the refusal of the family to give informed consent. No case had to be excluded due to anomalies or congenital conditions incompatible with life. In 4 cases, the family refused to give informed consent for inclusion in this study (those were all term neonates).

The data gathered were stored in a secure, blinded database on a firewalled computer, not connected to any network (internal or internet). The data can be obtained upon reasonable request after an email to adrian.toma@prof.utm.ro.

### 2.1. Ultrasound Technique and Measurements

The head ultrasound examination was performed between 48 and 72 h by a trained, experienced ultrasonographer [A.I.T.].

For the examinations, we used, in all cases, a Vivid S60 Ultrasound Machine (Manufacturer, General Electric HealthCare, Waukesha, West Milwaukee and Madison, WI, USA). A linear probe was used, with a frequency of 7–12 MHz. The colour Doppler and power Doppler modes were used to identify the vessels. To maintain the safety of the examination, the mechanical index (MI) and thermal index (TI) were maintained below 1.0 [26,27]. In the cases when we identified an atypical vascularisation pattern (see below), we used a Logiq E10 Ultrasound Machine (Manufacturer, General Electric HealthCare, Waukesha, West Milwaukee and Madison, WI, USA) equipped with a microvascular imaging (MVI) device [28], which allowed better identification of the vessels with a low flow velocity.

The choice of a linear probe for the examination was based on the following:

- The structures investigated were closer to the fontanel, and to better visualise them, we needed a high-frequency probe. The frequency used was 7.5–10 Hz. Linear probes have higher frequencies than microconvex ones [25].
- A microconvex probe has the disadvantage of compressing the fontanel and potentially causing an increase in intracerebral pressure according to some authors [29,30], so we preferred the linear probe also because it is safer for the patient.

The measurements were performed in coronal sections through the anterior fontanel.

**Figure 1:**
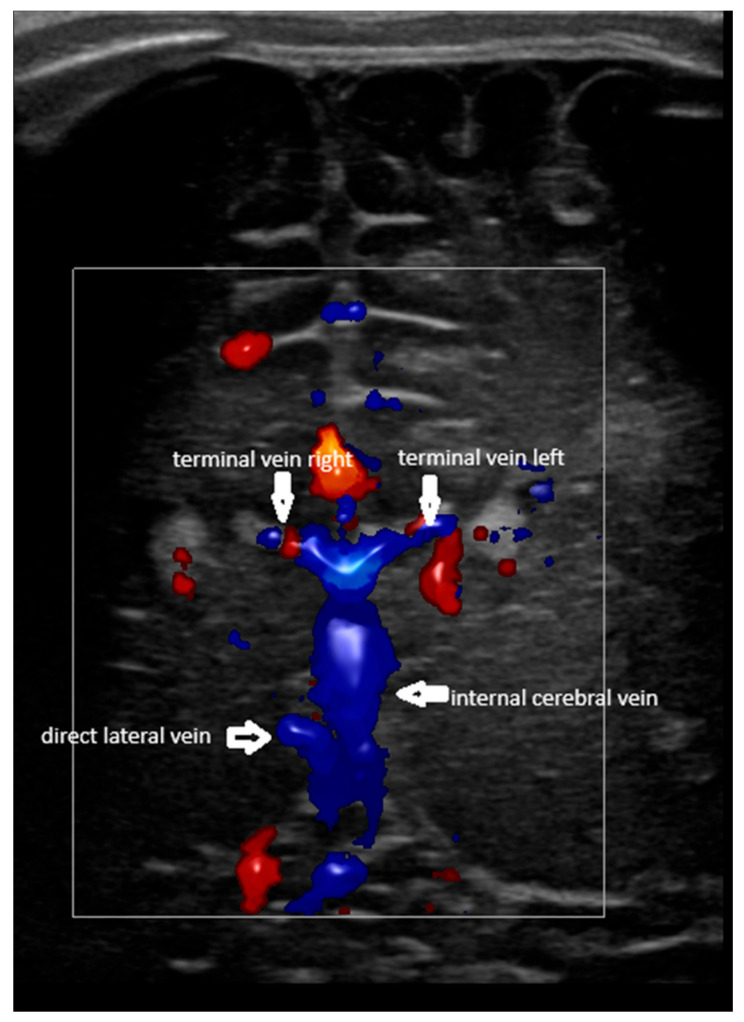
Coronal section; plan slightly posterior to the foramen of Monro. Colour Doppler. Normal pattern; confluence between the terminal (thalamostriate) veins and the internal cerebral vein identified. The direct lateral vein on the right is also visible on the right. The confluence is, in this case, slightly posterior to the foramen of Monro.

**Figure 2:**
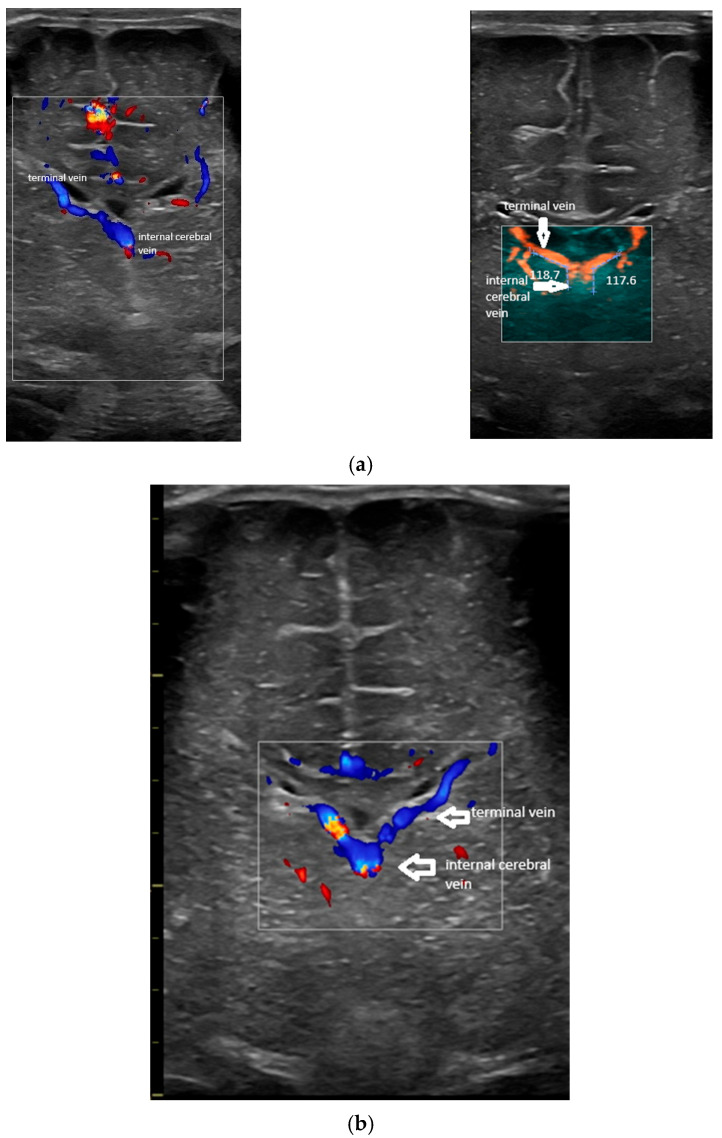
(**a**) Coronal section at the foramen of Monro (power Doppler). Normal pattern; confluence of the terminal vein and internal cerebral vein at the level of the foramen of Monro; patient with multicystic images in the caudo-thalamic groove, bilateral; GM/IVH grade 1 in evolution. (**b**) Colour Doppler (same section). The course of the terminal vein can be followed until the confluence with the internal cerebral vein.

**Figure 3:**
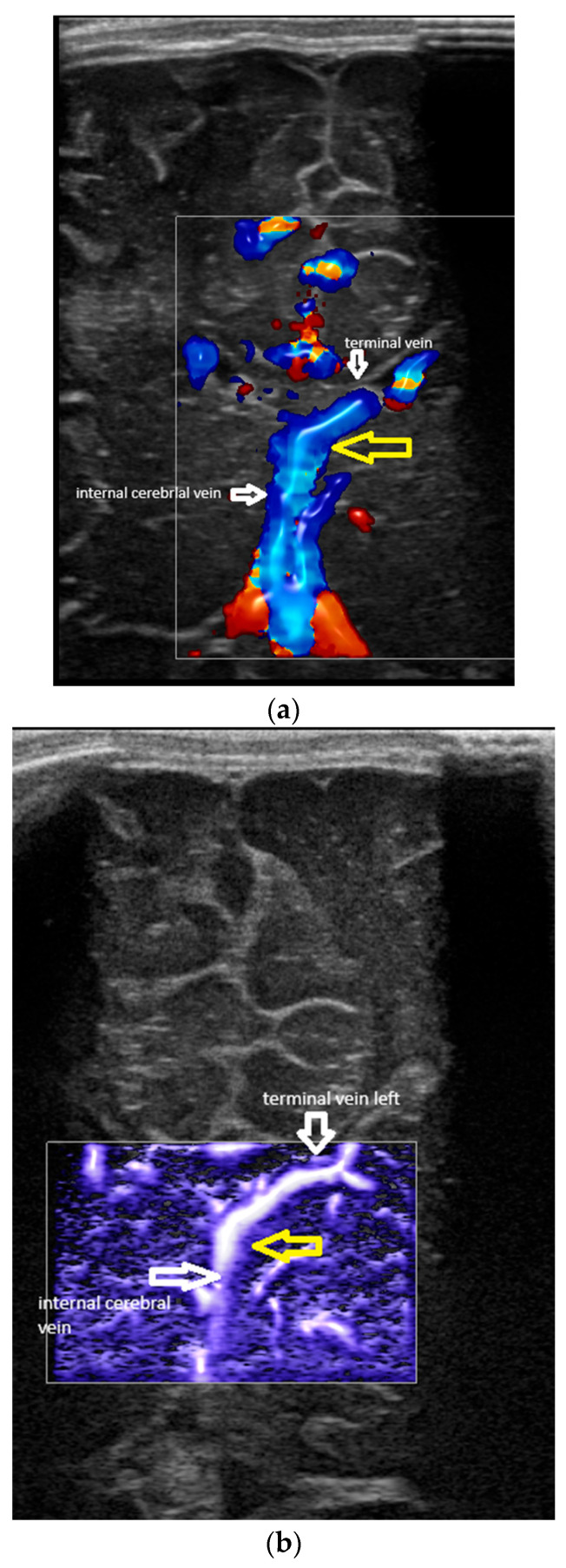

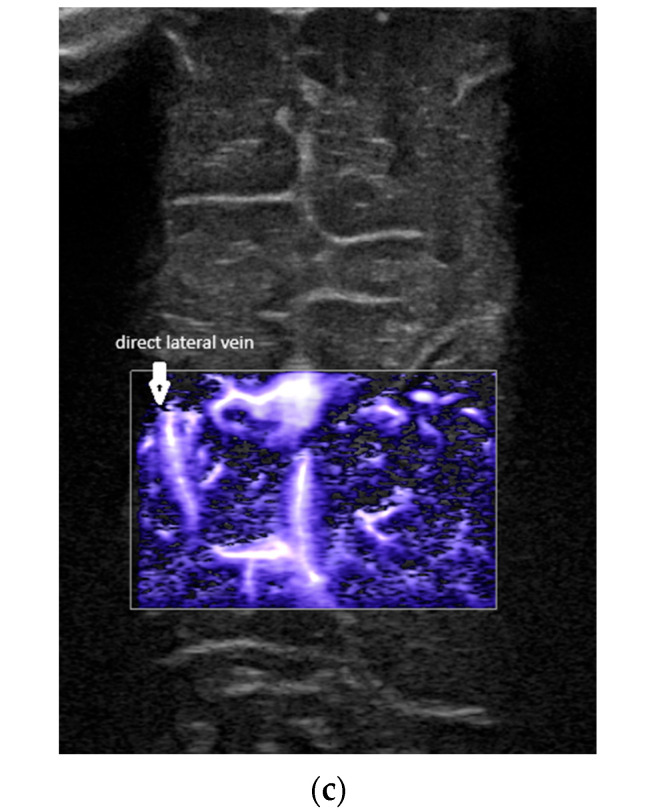
(**a**) Abnormal venous pattern (colour Doppler). The confluence between the terminal and the internal cerebral vein is identified only on the left (yellow arrow). (**b**) Abnormal venous pattern (microvascular imaging (MVI)); same view. Confluence between TV and ICV identified on the left only (yellow arrow). (**c**) Abnormal venous pattern (microvascular imaging (MVI)). A right direct lateral vein with an increased diameter can be observed on the side where the terminal vein is absent.

The terminal (thalamostriate) vein was first identified in a coronal section at the level of the foramen of Monro. The trajectory was parallel to the ventricular border, at the level of the head of the caudate nucleus. The place of the vein was classically identified by ultrasound [5]. Then, the probe was angulated slightly posteriorly in order to identify the confluence between the terminal and the internal cerebral vein. Based on the medical literature, a couple of situations were encountered [1,3,7,10]:

- Confluence posterior to the foramen of Monro, the most frequent variant (Figure 1).
- Confluence at the level of the foramen of Monro (Figure 2).
- A lack of identification of the confluence—wherein the terminal vein was absent on one or both sides—an example is the case presented in Figure 3, where the terminal vein was most probably draining in a direct lateral vein on the left side.

The identification of the confluence in the ultrasound was considered a normal variant; the absence of identification of the confluence was considered atypical.

When identified, the angle at the confluence between the terminal and internal cerebral vein was measured (Figure 2). The measurement was performed at the outer margin of the veins, on stored images, by two persons (A.I.T. and L.N.), blinded to other features of the case. In the case of discordance of the measurements, a third expert (R.B.) was consulted, and the mean of the measurements was considered for the database.

The following data were included in the database:

- The type of venous pattern (normal or atypical) on the left and right;
- The angles at the confluence of the terminal and internal cerebral vein on the left and right.

**Figure 4:**
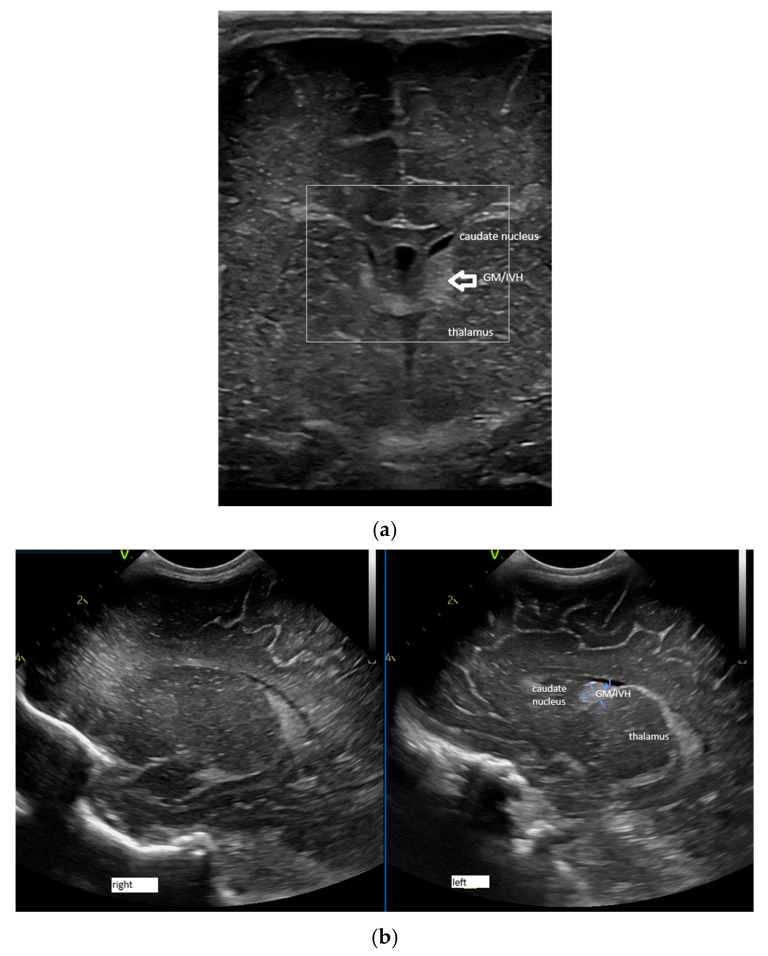
(**a**) GM/IVH (germinal matrix/intraventricular haemorrhage) grade 1 Volpe. Premature infant with a GA of 35 weeks (day of life). Coronal section; linear probe; GM/IVH identified as a hyperechogenic image (arrow) at the level of the caudothalamic groove. (**b**). Same patient (para-sagittal sections left and right); microconvex probe; GM/IVH can be observed as a hyperechogenic image at the level of the caudo-thalamic groove on the left.

The presence of a GM/IVH was noted in the ultrasound image (Figure 4) (identified as a hyperechogenic image at the level of the caudo-thalamic groove [25,31] and graded according to the Volpe classification [11]).

### 2.2. Rationale for Using Ultrasound: Advantages and Disadvantages

**Figure 5:**
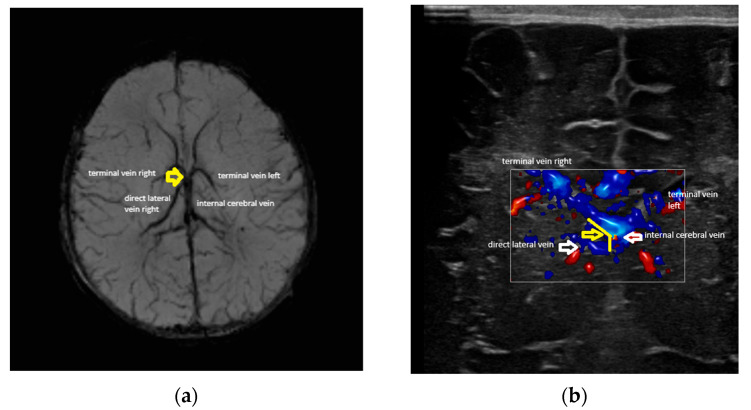
(**a**) MRI of deep brain venous system. (**b**) Colour Doppler (same patient).

As previously stated, the MRI exam, particularly SWI MRI, represents the gold standard for assessing the venous patterns of the deep venous system both in adults and neonates [1,4]. Not having an MRI control for the examinations represents the greatest flaw of our research. It is not possible to completely speculate how the data could be modified if MRI were used as a control. Several considerations could, though, be made. First, the plans that ultrasound and MRI investigate are different, and the angles measured by MRI and ultrasound are in different planes and of different values. Figure 5 shows the determination of the angle at the confluence between the terminal vein (TV) and the internal cerebral vein (IV), both in MRI and ultrasound. The SWI MRI sequence (Figure 5a) visualises the vessels and the confluence in a transverse plane (Figure 5a); the angle is shown by the red arrow. The ultrasound image (Figure 5b) represents a coronal plane; the angle is between the same vessels but in a different plane (yellow arrow). In conclusion, the angle between the two vessels is observed in both images, but in different planes.

Second, regarding the concordance between the MRI and ultrasound, we could not speculate about this sample, but a study published in 2007 showed a good concordance between the identification of the structures by MRI and head ultrasound in preterm neonates [31,32]. Nevertheless, this study investigated the brain structures, not the vessels, so the data are limited in this situation. In our case, an atypical venous pattern can be seen with the presence of a direct lateral vein draining at the confluence between the TV and ICV on the right side, but not on the left, in the images seen using both MRI and ultrasound.

Figure 5a,b show a comparison between MRI (SWI) and ultrasound (colour Doppler) in the same patient. The planes are different: transverse in the MRI (Figure 5a) and coronal in the ultrasound (Figure 5b). Consequently, the angles were different; they were between the same vessels (in this case, in the right terminal vein and the internal cerebral vein) but in different planes (the yellow arrow in Figure 5a,b). However, both images identified an anomaly: a direct lateral vein joining the confluence of the terminal and internal cerebral veins on the right, but not on the left.

The choice of ultrasound as the method for investigating the deep venous system was determined by our wish to use the method to develop preventive strategies in the future (obviously, we are not there yet, but it is a first step). The strategy would target the sickest and most fragile of neonates, and an imaging technique that requires (as described earlier for MRI) transport to a special place and immobilisation for a time interval could potentially harm these patients; instead, ultrasound can be performed safely in the sickest and most fragile patients, without moving them and with minimal stress. We consider, in the first place, this research to be a feasibility study, demonstrating that the assessment of the deep cerebral venous system is possible and determining the values and patterns in normal-term and preterm infants. Thus, even if found, statistically significant associations between angles, patterns, and GM/IVH will be regarded with caution.

### 2.3. Other Variables Studied

Other variables were also noted to better characterise the population studied: gestational age (weeks), the gender of the patient, birth weight, the status of the neonate (singleton or twins), the need for delivery room resuscitation, the presence of respiratory distress, the need for respiratory support, and the type of respiratory support (CPAP or mechanical ventilation).

### 2.4. Statistics

The statistical analysis was performed in another centre by a specialised statistician, provided with the database and the research question in order to avoid detection and reporting biases [33].

Testing for the normal distribution of the series of quantitative values was performed by using the Kolmogorov–Smirnov test used to analyse large samples of data [33]. The variables have a normal distribution if the level for testing the null hypothesis is *p* > 0.05. Skewness and kurtosis normality tests evaluate the distribution of a dataset that seems to have a normal distribution. The skewness test measures the asymmetry of the distribution, and the kurtosis test measures the homogeneity. We used these tests in the case of the measurements of the left and right angles at the confluence to establish if parametric tests could be applied that assumed a normal distribution [33,34].

The chi-square test was used in order to investigate if there was a correlation between the pattern of vascularisation (normal or atypical) and the category of neonates (born at term or preterm; singletons or twins), and between the normal or atypical venous patterns and the presence of GM/IVH. The chi-square test was also used in order to investigate the correlation between the angle at the confluence of the terminal and internal cerebral vein on the left and right and the presence of GM/IVH on that side. In order to assess the predictive value of the angles, we calculated the ROC curve for the angle of confluence [35].

The comparison between the values of the angle at confluence and different categories of neonates (born at term or preterm; singletons or twins; male or female gender) was evaluated by using a *t*-test for independent samples [35].

## 3. Results

A sample of 100 neonates was analysed in this study. Thirty-two premature neonates and sixty-eight neonates born at term were included. The characteristics of the population are presented in Table 1. As can be observed, there was a predominance of males in the premature infants group, and, as expected, more premature infants received respiratory support (CPAP or mechanical ventilation). All the patients survived to discharge; there was no death in either of the groups.

The mean gestational age of the group was 37.04 + 2.37 weeks (median: 38 weeks). There were seven pairs of twins, all di-zygotic and all born before 37 weeks of age.

### 3.1. Venous Drainage Patterns

An atypical venous drainage pattern was noted in 23 (33.8%) of the term neonates and 12 (37.5%) of the premature infants; there was no statistically significant difference between the groups (*p* < 0.443) (Table 2). Also, there was no statistically significant difference between twins and singletons regarding the venous drainage pattern (*p* < 0.413).

### 3.2. Angle at Confluence Between Terminal Vein and Internal Cerebral Vein

The statistical analysis showed that the values determined for the angles at the confluence between the terminal and the internal cerebral vein had a normal distribution both on the left (with variations between 110.9 and 159.3°; the mean of the group being 135.95 ± 11.37; and the median being 136.90) and the right (with variations between 105.60 and 154.50; the mean of the group being 138.16 ± 10.22; and the median being 138.20) (Table 3).

The values of the angles did not differ significantly between the term and preterm neonates (*p* < 0.279—left; *p* < 0.718—right) (Table 4). No statistically significant gender difference (*p* < 0.643—left; *p* < 0.399—right) (Table 5) or a difference between singletons and twins (*p* < 0.745—left; *p* < 0.418—right) was noted (Table 6).

**Figure 6:**
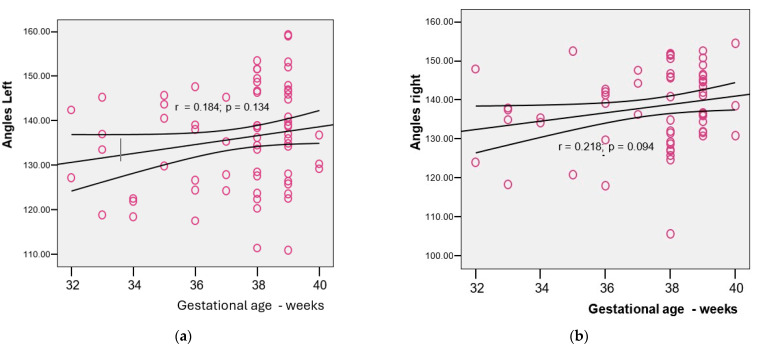
Correlation between the gestational age and the angle at the confluence of the terminal vein/internal cerebral vein ((**a**)—angle on the left; (**b**)—angle on the right).

There was no statistically significant correlation between the gestational age and the angles on the left (Figure 6a) or right (Figure 6b).

There was no statistically significant difference between the angles on the left and on the right in the whole group (*p* < 0.121) and the subgroups of term (*p* < 0.440) and preterm neonates (*p* < 0.092) (Table 7).

Regarding the situation in which the angle at the confluence could not be determined, on the left side, there were significantly more cases in the preterm than in the term neonates (*p* < 0.026); on the right side, there was no statistically significant difference between the groups (Table 8).

### 3.3. Correlations Between Venous Patterns and Angles and Risk of Intraventricular Haemorrhage in Premature Neonates

There was no statistically significant difference between the type of venous pattern and the occurrence of GM/IVH on the left (*p* < 0.344) or right (*p* < 0.485) in the subgroup of preterm neonates (Table 9).

**Figure 7:**
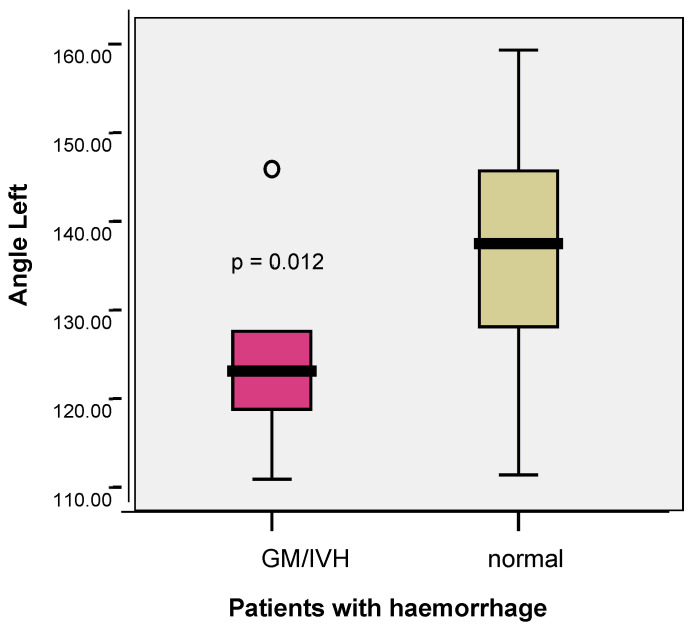
Comparison between the mean value of the angle on the left and the risk of GM/IVH.

The mean value of the angle at the confluence between the terminal vein and the internal cerebral vein on the left was significantly lower in the premature infants with an ultrasound image of the left GM/IVH than in the premature neonates without a haemorrhage (124.90 vs. 137.02; *p* = 0.012) (Figure 7). The angle could not be determined in the patients with right GM/IVH.

**Figure 8:**
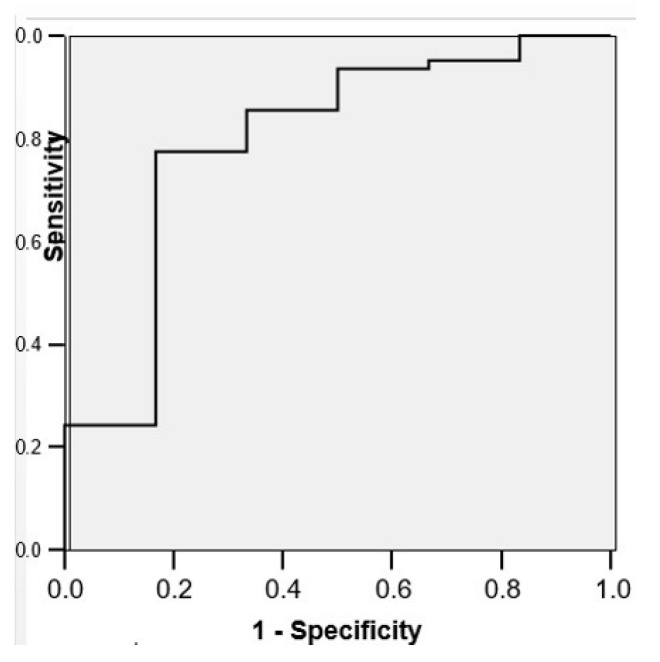
The specificity/sensitivity balance of the angle at the confluence on the left and the occurrence of GM/IVH.

The ROC curve confirmed the fact that the angle at the confluence could be a predictor for GM/IVH (AUC = 0.793; CI 95%: 0.580–1.006; *p* = 0.018), with a sensitivity of 79%, a specificity of 67%, and a cut-off value of 126.90 (Figure 8).

**Figure 9:**
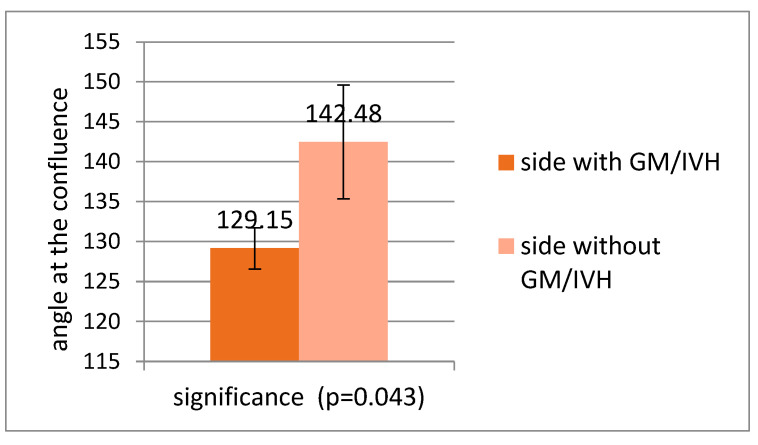
Comparison of the angles of confluence on the side with haemorrhage and without haemorrhage in patients with GM/IVH.

In patients with GM/IVH, the angle was significantly lower on the side with haemorrhage than on the side without haemorrhage (*p* < 0.043) (Figure 9).

## 4. Discussion

Our research showed that, in the case of the deep cerebral venous system, particularly in the medullary–thalamostriate–internal cerebral vein system, the configuration and the angles of confluence could be identified by Doppler ultrasound examination in term and preterm neonates. The angles and the pattern of venous drainage were not different between the term and moderate-to-late preterm neonates. The finding that the angle of confluence between the terminal (thalamostriate) and internal cerebral vein was associated with the occurrence of GM/IVH on the side with a tighter angle should be regarded with caution due to several factors, and especially the small number of cases.

We are aware of certain weak points in our study. The first and most important is the lack of a comparison with the MRI images, which represent the gold standard in the visualisation and analysis of the deep cerebral venous system in neonates [1]. Indeed, there are studies that have assessed the structure of the neonatal deep cerebral venous system by using MRI SWI venography [3,4]. The choice of ultrasound (in this case, Doppler ultrasound) carries many problems related to different factors. First, the plans obtained by MRI venography are transverse and allow the visualisation of all the vessels in one image [3,4]. The plans obtained by ultrasound are perpendicular and oblique to the MRI plans [24,25]. They allow the visualisation of most of the vessels by Doppler [7,24], but several plans/images are necessary in order to obtain a good estimate of the structures. Also, Doppler imaging is operator-dependent, and it is not considered good to assess the cerebral blood flow, though the most recent review finds a good place for Doppler imaging in assessing vascular brain structures in order to detect malformations [24]. Our choice for this technique was supported by its ease of performance, the fact that it was not necessary to move the patient to the MRI department, and the short duration of the investigation. Indeed, we plan to use MRI comparators in a future study to validate the findings. To overcome the operator dependency of the measurements, we allowed three persons to perform the measurements and developed a protocol to avoid and harmonise the discordances (see the Methods Section)

Another weak point was the fact that we did not separate the venous drainage patterns in the six categories previously described for neonates [3,4]. As has already been described above, the categories were separated by the presence/absence of the terminal (thalamostriate) vein, the confluence at or posterior to the foramen of Monro between that vein and the internal cerebral vein, and the presence or absence of the direct lateral vein and the anterior septal vein [3]. The use of ultrasound did not allow for the exact determination of these categories, though we could assess if a terminal vein was present, if there was a confluence between the terminal and the internal cerebral veins and if there were other veins that drained into the internal cerebral vein. It should be noted, though, that one of the objectives of our study was to identify vascular patterns that could represent a risk factor for the occurrence of GM/IVH, and since the vein involved was the terminal vein and due to its confluence with the internal cerebral vein [11,12,26], identifying the variation in these structures could be a good start to identify infants at risk. The small number of cases did not allow the building of a multivariate analysis model taking into account other variables, which could also be considered a weakness of this study.

Due to the small number of cases, the findings regarding the association of the patterns and angles with GM/IVH should not be overinterpreted and should be regarded with caution. Thus, the findings regarding the association of GM/IVH with a certain angle are exposed to the risk of type 1 and 2 statistical errors [34,35]. Type 1 error—i.e., we wrongly reject the null hypothesis, and there is no correlation between the angle at the confluence of the TV and ICV—carries the risk of erroneously identifying infants as at risk and applying prevention strategies based on this assumption. Even if the *p*-value is low (<0.012), the risk is present, and the findings should be regarded with caution. However, a type 2 error in this case—accepting the null hypothesis and affirming that there is no link between the angles and the occurrence of IVH—could also be dangerous in erroneously considering infants who are at risk for GM/IVH to be at no risk. This error is also possible due to the same factor: a small number of cases. It should be stated, then, that since the incidence of the pathology is low (as could be seen in the Introduction), a large multicentric study is needed to assess the validity of the findings in this pilot study and to strengthen the power of the associations, if any are found.

Concerning the finding that a narrower angle at the confluence was found in the cases with GM/IVH on the left but not on the right, this could be a result of statistical scatter. Thus, several considerations regarding this asymmetry could be mentioned:

- In the medical literature, a slight left-sided predominance of IVH is noted. It should be mentioned that the studies did not look for this particular feature. In the study of Tortora and co-workers comparing the angles between the internal cerebral vein and the thalamostriate vein [4], a slight predominance of haemorrhage on the left was noticed, with 8/14 cases of unilateral haemorrhage occurring on the left. Also, in our database, the number of IVH cases was double on the left compared with the right.
- As is already known from the medical literature, certain cerebrovascular diseases of neonates (arterial ischemic stroke) occur more often on the left than on the right due to the hemodynamic particularities of the neonatal circulation [18]. Although the asymmetries in the arterial system of the brain are well known and associated with pathologies, the venous system of the brain also presents with asymmetries of structure and calibre, especially on the left, due to developmental particularities [36]. We could speculate that these structural particularities could play a role in the occurrence of IVH more on the left than on the right, and that the vascular factors are more involved in the appearance of this lesion.
- Several of the components of the bundle of care for the prevention of GM/IVH in premature infants are related to venous return and, particularly, to avoidance of increased venous pressure (maintaining the head on the midline and the avoidance of placing the head lower than the body position [37,38]), a fact underlying, once more, the importance of venous factors in the appearance and progression of the lesion.

We consider that there were also strong points of our research. The first is the novelty of the approach. There is no other study, to our knowledge, addressing this issue of the angle measured by ultrasound (the other studies we know addressed this issue on another angle, measured by MRI [3,4]). Also, even if there was consensus about the possibility of identifying most of the vessels forming the deep cerebral venous system [5,7], our approach is systematic and offers a frame of normal values that would guide further research. Also, besides the use of classic Doppler modes—colour and power Doppler—we also used new Doppler techniques (microvascular imaging (MVI)) in order to obtain clarifications in certain cases. Another strong point is the protocol used by us in order to avoid detection and reporting biases (see above).

Our research had two components: first, we established a set of normal values for the angles at the confluence and venous drainage patterns in different categories of neonates, and then, we assessed if, based on these patterns and angles, a group of premature infants at risk for the occurrence of GM/IVH could be identified. The decision to include term neonates in our research was based on the fact that we did not find a study in the literature to determine angles at the confluence between the terminal and the internal cerebral vein measured by ultrasound (the other study determined another angle and was performed on MRI images) [4]. We had to determine the normal values and appearances in term neonates in order to better characterise these parameters in preterm neonates and to assess if there were significant differences related to the gestational age.

This research did not find any statistically significant difference between term and preterm neonates, singletons and twins, or male or female neonates regarding the angle at the confluence between the TV and ICV or the presence of a normal or atypical venous pattern. This situation is contrary to the one presented in other studies using MRI as a method to identify the angles and a more rigorous method of calculating the value of the curvature [3,4]. This represents another important weakness of our research to be acknowledged. There could be, though, a couple of points to discuss related to this. First, the study cited identified six types of deep medullary venous systems, and our study identified just the pattern to be a normal TV/ICV junction present, which could be types 1, 2, and 5 in the above-mentioned study, or an atypical TV/ICV junction absent, corresponding to types 3.4 and 6 [3]. Second, the previous study classified the premature infants into two categories: very preterm neonates (gestational age < 32 weeks) and moderate-to-late preterm neonates (32–36 weeks) [3]. Our population, as could be seen from the tables and graphs, consisted mainly of premature infants > 32 weeks and moderate–late preterm infants. In this case, the situation of the patterns in the moderate-to-late preterm infants was more similar to that in the term neonates [3]; in addition, the same patterns were not compared.

Regarding the angle at the confluence, the angle that is investigated by ultrasound is different from the angle that is measured by MRI [4,5,24]. We did not find any statistically significant difference between the different categories of the above-mentioned neonates. Also, there was no difference between the angles on the left and those on the right, both in term and preterm neonates. The reason for this is also related to the population studied. As is known from the literature, the variability in the deep medullary venous system is, at least in part, explained by the way the system is formed by the re-configuration of the venous plexuses during the fetal period [37,39,40,41,42]. It is speculated that, in a premature infant, the system is immature and has not yet attained its final form, and the relatively hyperoxic extra-uterine environment determines the deficient formation of the structures [3,4]. A difference has also been described in the development of the vascular structures of the two hemispheres [3,43,44]. We could speculate, though, that the venous system is more mature and the side-related differences are less important in moderate–late preterm infants, which were predominant in our study group, than in very preterm neonates, and that could be the reason for not noticing any differences regarding the pattern and angles.

We also identified a correlation between the angle at the confluence between the TV and ICV and the presence of GM-IVH. In addition, a significantly smaller angle could be identified in a certain patient, on the side on which the haemorrhage would occur (the angle on the side with the GM/IVH is significantly smaller than on the side without). As mentioned above, we recommend considering this finding with caution and avoiding over-interpreting, especially due to the small number of cases. This finding underlines the importance of vascular factors in the occurrence of GM/IVH in neonates. Indeed, even if the pathology of GM/IVH is complex and involves several types of factors [11], since the first description of the lesion, a very important role has been attributed to the particular vascular structure involved, and especially to the U-turn of the terminal vein at the confluence with the ICV [12]. Considering the venous origin of the lesion [3], the angle at the confluence becomes more important because a tighter angle would favour venous stasis and increase the risk for the haemorrhage [11,12]. Other groups have also considered vascular venous factors, in particular, increased venous pressure. Two of the interventions in the first published bundle of care [39] aimed at decreasing venous stasis: maintaining the head on the midline to avoid stenosis/closure of the jugular veins and the avoidance of the Trendelenburg position to facilitate gravitational venous drainage. We believe that early identification of a tighter angle at the confluence of the TV/ICV could select a population at risk for applying supplementary interventions in the bundle of care in order to prevent the appearance of GM/IVH. Screening strategies have been used in neonates for the early identification of hearing impairment [45] and metabolic or endocrine anomalies [46,47] to establish prevention or early treatment strategies. The sensitivity and specificity of the cut-off value are quite low, though the specificity (67%) is lower than the sensitivity (79%), so the test will better identify individuals at risk than those with a lower risk for GM/IVH [35].

Thus, the association is weak, and caution should be used to not over-interpret it, to avoid resulting in a significant number of false positives and overtreating infants that are not truly at risk and producing unjustified anxiety in the families.

Due to many factors related to the technique used (ultrasound and not MRI, the difficulty of measuring the angles and patterns, and the lack of an MRI comparison), unicentric design, and population examined (moderate-to-late preterm and term neonates), the results of this study should be regarded with caution and need validation in multi-centric research, also using MRI as a comparator and with a population containing both small and very small premature neonates.

## 5. Conclusions

The vascular pattern of the deep cerebral venous system and the angle at the confluence between the terminal vein and the internal cerebral vein could be identified in term and preterm neonates by Doppler ultrasound. We found no difference in our group in the central venous pattern or angle at the confluence of the TV and the ICV between different categories of neonates. The finding that the angle at the confluence between the TV and ICV could identify cases at risk for GM/IVH as well as the side of occurrence of the haemorrhage, offering the opportunity of developing personalised prevention strategies, should be regarded with caution due to the small number of cases, the weak specificity and sensitivity, and the population studied, which does not represent the main group at risk, and more multicentric investigations are needed to test this hypothesis in a larger number of cases of different gestational ages.

## Figures and Tables

**Table 1:** Characteristics of the population studied.

	Preterm	Term	*p*-Value
Number	32	68	NA
Gestational age—weeks (+standard deviation)	34.53 (+2.42)	38.22 (+1.09)	0.001
Birth weight—grams (+standard deviation)	2372 (+639.91)	3180 (+426.24)	0.001
Male	24/32	35/68	0.087
Female	8/32	33/68	
CPAP	25/32	1/68	0.001
Administration of oxygen	30/32	11/68	0.004
Hypoglycaemia	10/32	12/68	0.103
Mechanical ventilation	7/32	2/68	0.004

**Table 2:** Correlation between the venous drainage pattern and the type of neonate.

Category of Neonates	Venous Drainage Pattern		Total	Chi2 Test *p*
	Atypical (*n* = 35)	Normal (*n* = 65)		
Term	23 (65.7%)	45 (69.2%)	68 (68.0%)	0.443
Preterm	12 (34.3%)	20 (30.8%)	32 (32.0%)	
Twins	4 (11.4%)	10 (15.4%)	14 (14.0%)	0.413
Singletons	31 (88.6%)	55 (84.6%)	86 (86.0%)	

**Table 3:** Descriptive statistical indicators of the angles at the confluence—left and right.

Number of Cases		Left	Right
		68	60
Mean (degrees)		135.95	138.16
Median (degrees)		136.90	138.20
Standard deviation (degrees).		11.37	10.22
Variance		8.36	7.40
Kurtosis test		−0.659	0.450
Kurtosis standard error		0.574	0.608
Kolmogorov–Smirnov		0.200	0.200
Minimum		110.90	105.60
Maximum		159.30	154.50
Percentiles	25	126.75	131.53
	50	136.90	138.20
	75	145.60	146.13

**Table 4:** Comparison between term and preterm neonates (angles at confluence).

	Number	Mean	Standard Deviation	Standard Error of Mean	Min.	Max.	*t*-Test for Independent Samples	
							*T*	*p*
**Angles—Left**:								
Term	51	136.82	11.44	1.602	110.90	159.30	1.189	0.279
Preterm	17	133.35	11.05	2.681	117.50	151.60		
Total	68	135.95	11.37	1.378	110.90	159.30		
**Angles—Right**:								
Term	44	138.45	10.26	1.547	105.60	154.50	0.132	0.718
Preterm	16	137.36	10.39	2.598	118.00	152.50		
Total	60	138.16	10.22	1.319	105.60	154.50		

**Table 5:** Comparison between genders (angles at confluence).

	Number	Mean	Standard Deviation	Standard Error of Mean	Min.	Max.	*t*-Test for Independent Samples	
							*t*	*p*
**Angles—Left**:								
Male	40	136.49	11.49	1.816	111.40	159.30	0.216	0.643
Female	28	135.18	11.36	2.146	110.90	159.00		
Total	68	135.95	11.37	1.378	110.90	159.30		
**Angles—Right**:								
Male	37	139.05	10.18	1.673	118.00	152.60	0.721	0.399
Female	23	136.74	10.34	2.157	105.60	154.50		
Total	60	138.16	10.22	1.319	105.60	154.50		

**Table 6:** Comparison between singletons and twins.

	Number	Mean	Standard Deviation	Standard Error of Mean	Min.	Max.	*t*-Test for Independent Samples	
							*t*	*p*
**Angles—Left**:								
Twins	7	134.61	10.00	3.780	121.90	145.70	0.107	0.745
Singletons	61	136.11	11.58	1.482	110.90	159.30		
Total	68	135.95	11.37	1.378	110.90	159.30		
**Angles—Right**:								
Twins	8	135.41	7.41	2.619	120.80	142.70	0.665	0.418
Singletons	52	138.59	10.58	1.467	105.60	154.50		
Total	60	138.16	10.22	1.319	105.60	154.50		

**Table 7:** Comparison of the angles on the left and right between the two groups.

Category	Left (Mean + SD)	Right (Mean + SD)	*t*-Test for Independent Samples
Total	135.9 (+10.2)	139.06 (+15.8)	0.121
Term neonates	133.37 (+8.4)	139.26 (+12.8)	0.440
Preterm neonates	133.95 (+9.6)	138.37 (+15.4)	0.092

**Table 8:** Comparison of the cases in which the angle could not be determined between term and preterm neonates (left and right).

Side	Number of Cases with an Unidentified Angle	Neonates		Chi2 Test *p*
		Term (*n* = 68)	Preterm (*n* = 32)	
Left	32	17 (25.0%)	15 (46.9%)	0.026
Right	40	24 (35.3%)	16 (50.0%)	0.119

**Table 9:** Correlation between the venous pattern and the occurrence of GM/IVH in the group of premature neonates.

GM/IVH	Venous Pattern		Total	Chi2 Test *p*
	Atypical (*n* = 12)	Normal (*n* = 20)		
Left	2 (16.7%)	6 (30.0%)	8 (25.0%)	0.344
Right	2 (16.7%)	2 (10.0%)	4 (12.5%)	0.485

## Data Availability

The database of this study can be accessed upon request at the following address: adrian.toma@prof.utm.ro.

## Abbreviations

The following abbreviations were used in this manuscript:

CI	Confidence interval
CPAP	Continuous positive airway pressure
GM/IVH	Germinal matrix/intraventricular haemorrhage
ICV	Internal cerebral vein
MI	Mechanical index
MRI	Magnetic resonance imaging
MVI	Microvascular imaging
ROC curve	Receiving operating characteristic curve
TV	Terminal vein
TI	Thermal index
